# Changes in α-Synuclein Posttranslational Modifications in an AAV-Based Mouse Model of Parkinson’s Disease

**DOI:** 10.3390/ijms241713435

**Published:** 2023-08-30

**Authors:** Viviana Brembati, Gaia Faustini, Francesca Longhena, Tiago Fleming Outeiro, Arianna Bellucci

**Affiliations:** 1 Department of Molecular and Translational Medicine, University of Brescia, Viale Europa 11, 25123 Brescia, Italyfrancesca.longhena@unibs.it (F.L.);; 2 Department of Experimental Neurodegeneration, Center for Biostructural Imaging of Neurodegeneration, University Medical Center Goettingen, 37073 Goettingen, Germany; 3 Max Planck Institute for Multidisciplinary Sciences, 37075 Goettingen, Germany; 4 Translational and Clinical Research Institute, Faculty of Medical Sciences, Newcastle University, Framlington Place, Newcastle Upon Tyne NE2 4HH, UK

**Keywords:** Parkinson’s disease, alpha-synuclein, posttranslational modifications, phosphorylation, nitration, acetylation

## Abstract

Parkinson’s disease (PD) pathology is characterized by the loss of dopaminergic neurons of the nigrostriatal system and accumulation of Lewy bodies (LB) and Lewy neurites (LN), inclusions mainly composed of alpha-synuclein (α-Syn) fibrils. Studies linking the occurrence of mutations and multiplications of the α-Syn gene (*SNCA*) to the onset of PD support that α-Syn deposition may play a causal role in the disease, in line with the hypothesis that disease progression may correlate with the spreading of LB pathology in the brain. Interestingly, LB accumulate posttranslationally modified forms of α-Syn, suggesting that α-Syn posttranslational modifications impinge on α-Syn aggregation and/or toxicity. Here, we aimed at investigating changes in α-Syn phosphorylation, nitration and acetylation in mice subjected to nigral stereotaxic injections of adeno-associated viral vectors inducing overexpression of human α-Syn (AAV-hα-Syn), that model genetic PD with *SNCA* multiplications. We detected a mild increase of serine (Ser) 129 phosphorylated α-Syn in the substantia nigra (SN) of AAV-hα-Syn-injected mice in spite of the previously described marked accumulation of this PTM in the striatum. Following AAV-hα-Syn injection, tyrosine (Tyr) 125/136 nitrated α-Syn accumulation in the absence of general 3-nitrotirosine (3NT) or nitrated-Tyr39 α-Syn changes and augmented protein acetylation abundantly overlapping with α-Syn immunopositivity were also detected.

## 1. Introduction

Parkinson’s disease (PD) is the second most common neurodegenerative disorder, affecting 2% of the population over 65 years of age [1]. A key neuropathological hallmark of PD is the deposition of intracytoplasmic inclusions mainly composed of alpha synuclein (α-Syn) in cell bodies and neurites [2], which are called Lewy bodies (LB) and Lewy neurites (LN), respectively [3]. In addition to nigrostriatal dopaminergic neurons degeneration, the presence of LB constitutes a confirmative post-mortem diagnostic marker for the diagnosis of PD [4]. 

Several lines of evidence support that the pathological deposition of α-Syn insoluble inclusions in the brain of patients affected by PD plays a causative role in neurodegeneration. For instance, among the most severe rare autosomal dominant genetic forms of PD there are those associated with multiplications in the α-Syn gene locus (*SNCA*) [5]. In addition, the spreading of LB pathology in the brain of PD patients appears to correlate with disease progression [6]. 

α-Syn is a 14kDa protein composed of 140 amino acids and predominantly expressed at presynaptic terminals in different regions of the central nervous system (CNS) [7,8]. Interestingly, several posttranslational modifications (PTMs) of α-Syn have been found to increase or decrease α-Syn aggregation, depending on the PTM [9]. Indeed, they can affect α-Syn aggregation propensity, solubility and turnover, membrane binding and interaction with other proteins and metals [9,10,11,12,13,14]. Phosphorylation, nitration, acetylation, ubiquitination, SUMOylation, O-GlcNAcylation and glycation are the most studied PTMs and may occur in the pathogenesis of PD [15].

In particular, α-Syn can be phosphorylated at serine (Ser87 and Ser129) or tyrosine (Tyr125, Tyr133 and Tyr136) residues [15]. Among them, Ser129 is the most studied α-Syn PTM, as it is considered a marker of mature fibrillary aggregates [16,17]. This PTM also controls aggregates formation [18,19,20] and influences α-Syn toxicity [21]. Moreover, recent evidence supports that phosphorylation at Ser129 controls also the physiological function of α-Syn [22,23,24]. 

Interestingly, it has been found that oxidative stress (OS) is strictly involved in α-Syn aggregation as it occurs with aging, which is the major risk factor for sporadic PD, and markedly contributes to the degeneration of dopaminergic neurons [25]. Protein nitration is considered a marker of oxidative damage and nitrated α-Syn can be easily formed under OS conditions and is also found in LB [26,27]. The presence of peroxynitrite induces the deposition of 3-nitrotyrosines (3NT) and the formation of 3,3-dityrosine via the oxidation of Tyr residues, thus promoting α-Syn oligomer formation [28]. Though we know that the sites of α-Syn nitration are Tyr-39, 125, 133 and 136, the impact of nitration on α-Syn aggregation is not fully understood as some evidence supports that nitrated α-Syn monomers and dimers can accelerate fibril formation [29], while other reports showed that nitration inhibits α-Syn fibrillation by inducing the formation of soluble oligomers [30].

One of the major PTMs found in eukaryotes is acetylation, mostly known for its role in transcriptional regulation. In humans, 80–90% of all proteins are acetylated at their N-termini (Nt) predominantly on methionine, alanine and serine [31,32,33] in an irreversible way, stabilizing α-helical structures in both proteins and peptides [34]. Of note, all the in vivo detectable α-Syn is posttranslationally modified by an acetyl group attached to the amino group of the first N-terminal amino acid [35,36,37]. Nt-acetylated α-Syn is resistant to amyloid aggregation, enhancing both protein-protein and protein-membrane interaction [38]. The deacetylation of histones operated by histone deacetylase (HDAC) is also implicated in the control of α-Syn toxicity as it plays a key role in the clearance of misfolded and aggregated protein [39,40,41,42,43]. Interestingly, sirtuin 1 (SIRT1), a nicotinamide adenine dinucleotide (NAD+)-dependent HDAC, has been found to exert neuroprotective effects [44] and mitigates α-Syn pathology through the induction of the chaperone heat shock protein 70, which prevents the misfolding or clears the aggregates by promoting degradation [45]. Furthermore, α-Syn aggregation propensity is controlled by lysine (Lys) acetylation [46,47,48,49,50]. For instance, it has been found that acetylated α-Syn and acetylated α-tubulin inhibit oligomer formation [51]. Moreover, Sirtuin 2 (SIRT2) deacetylates α-Syn and the inhibition of SIRT2 decreases α-Syn toxicity [49,52]. α-Syn PTMs, thus, appear to exert a key role in the pathogenesis of PD.

In this study, we aimed at investigating whether the increased expression of human α-Syn that characterizes genetic forms of PD associated with *SNCA* gene multiplications induces α-Syn nitration and acetylation in addition to the accumulation of Ser129-phosphorylated that is a marker of mature aggregates. In particular, the overexpression of human full length α-Syn in the nigrostriatal system of C57BL/6J mice was induced by using adeno associated viral vectors (AAV) as previously described [53,54,55,56] and we evaluated the occurrence of Ser129 phosphorylation, Tyr39, Tyr125 and Tyr136 nitration as well as of α-Syn acetylation by immunofluorescence (IF) and confocal microscopy analysis of the substantia nigra (SN) and striatum. We found that increased expression of α-Syn is accompanied by a specific subset of α-Syn PTMs.

## 2. Results

### 2.1. AAV-Based hα-Syn Overexpression Reproduces PD-like Neuropathological Features in C57BL/6J Wild Type Mice

The AAV-mediated overexpression of α-Syn in the nigrostriatal system of mice or rats is considered a valuable method to reproduce genetic forms of PD associated with SNCA multiplications [57]. Consistently, the AAV-mediated overexpression models have been found to exhibit PD-like alterations such as the formation of α-Syn cytoplasmic toxic inclusions, alterations in dopaminergic neurotransmission and progressive degeneration of the nigrostriatal system that are already detectable after only 8 weeks from the AAV inoculation [53,54,55,56,58,59].

**Figure 1:**
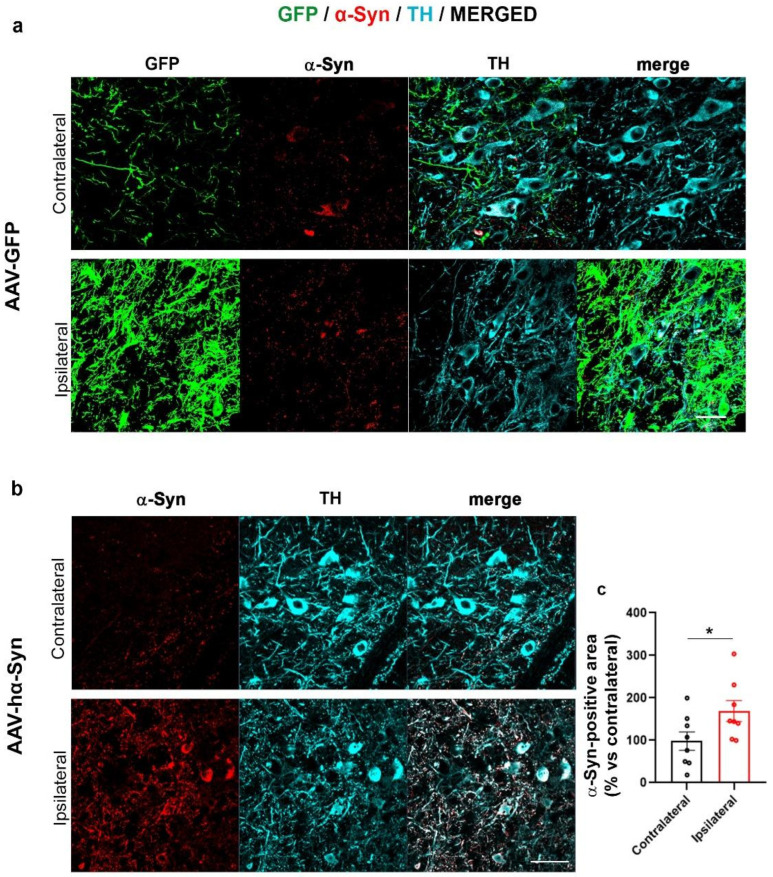
Increased α-Syn immunolabeling in the AAV-hα-Syn-injected SN. Representative images showing GFP signal and α-Syn-(red), and TH-(cyan) immunolabeling in SN of 4-month-old C57BL/6J mice injected with AAV-GFP (**a**) and AAV-hα-Syn (**b**) Scale bars: 20 μm. (**c**) The graph is showing the analysis of α-Syn-immunopositive area in the SN IL or CL to AAV-hα-Syn injection. A statistically significant increase in total α-Syn-immunopositive area of the IL compared to the CL hemisphere was detected (total area mean difference: +69.08%, * *p* < 0.05 unpaired Student’s *t*-test, N = 8).

**Figure 2:**
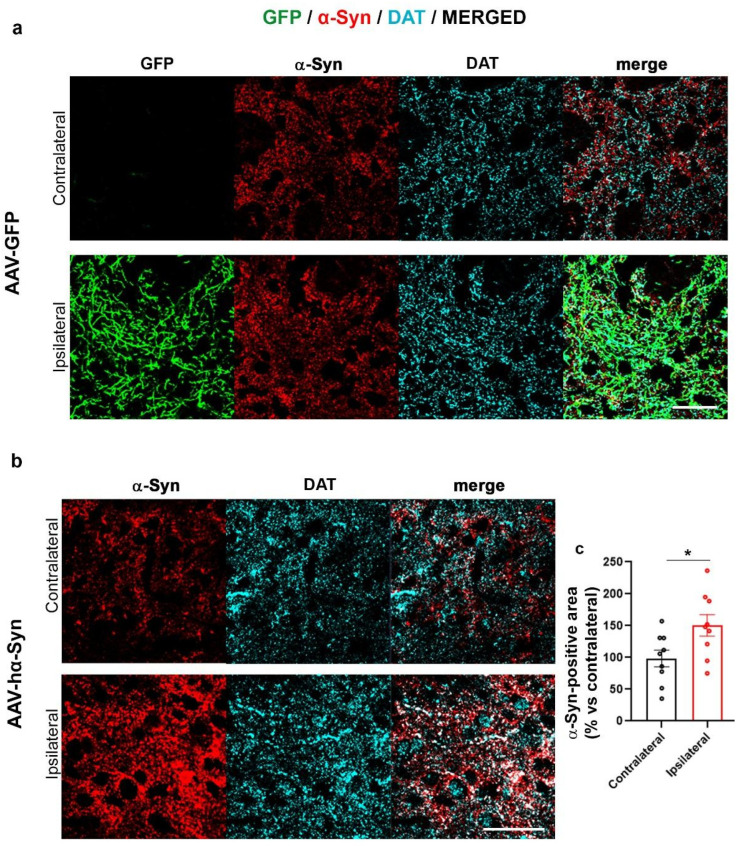
Increased α-Syn immunolabeling in the striatum IL to AAV-hα-Syn-injection. Representative images showing GFP signal and α-Syn-(red), and DAT-(cyan) immunolabeling in the striatum of 4-month-old wt mice injected with AAV-GFP (**a**) and AAV-hα-Syn (**b**) Scale bars: 20 μm. (**c**) The graph is showing the analysis of α-Syn-immunopositive area in the striatum IL and CL to AAV-hα-Syn injection. Please note the statistically significant increase in α-Syn-positive area in the striatum IL to AAV-hα-Syn injection vs. the CL side (total area mean difference: +50.41%, * *p* < 0.05 unpaired Student’s *t*-test, N = 9).

Here, we first checked whether the AAV-hα-Syn induced the overexpression of α-Syn and then we evaluated whether α-Syn deposits in the SN and striatum ipsilateral (IL) to the injection exhibit different PTMs. AAV-GFP injected mice were used as controls (Figure 1a and Figure 2a).

We found that the unilateral overexpression of AAV-GFP did not affect α-Syn levels, both in SN (Figure 1a) and striatum (Figure 2a) of C57BL/6J mice, in line with our previous observations [56]. The overexpression of hα-Syn induced the expected significant accumulation of α-Syn in the SN and striatum (Figure 1b and Figure 2b).

**Figure 3:**
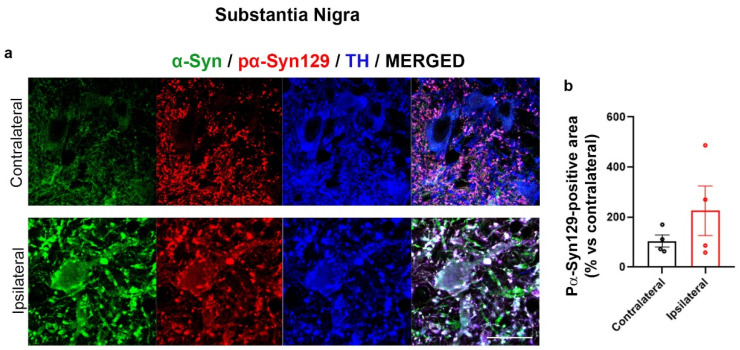
Mild increase in pα-Syn129 immunolabeling in the SN IL to AAV-hα-Syn injection. (**a**) Representative photomicrographs showing α-Syn-(green), pα-Syn129-(red) and TH-immunolabeling (blue) in the IL and CL SN of 4-month-old C57BL/6J mice injected with AAV-hα-Syn. Scale bar: 20 μm. (**b**) Graph is showing the analysis of pα-Syn129-immunopositive signal. No statistically significant difference was detected, N = 4.

As we already described that the striatum IL to AAV-hα-Syn injection of C57BL/6J wild type (wt) mice displays a marked immunopositivity for Ser129 phosphorylated α-Syn (pα-Syn129) [56], in the present study we only assessed the immunoreactivity for pα-Syn129 in the injected SN; we found that pα-Syn129 localized with α-Syn in a dot-like pattern in the cell body of TH-positive neurons (Figure 3a) and it exhibited an increase trend, that, however, was not statistically significant (Figure 3a,b).

These data supported that AAV-hα-Syn injection produced the expected increase in α-Syn as well as deposition of pα-Syn129. However, the lower pα-Syn129 accumulation observed in the SN, when compared to the previously described marked increase in pα-Syn129 detected in the striatum of AAV-hα-Syn-injected mice [56], suggests that this PTM principally occurs at neuronal terminals at least in the initial phases of PD.

### 2.2. Increased α-Syn Nitration in the AAV-hα-Syn-Based Mouse Model of PD

**Figure 4:**
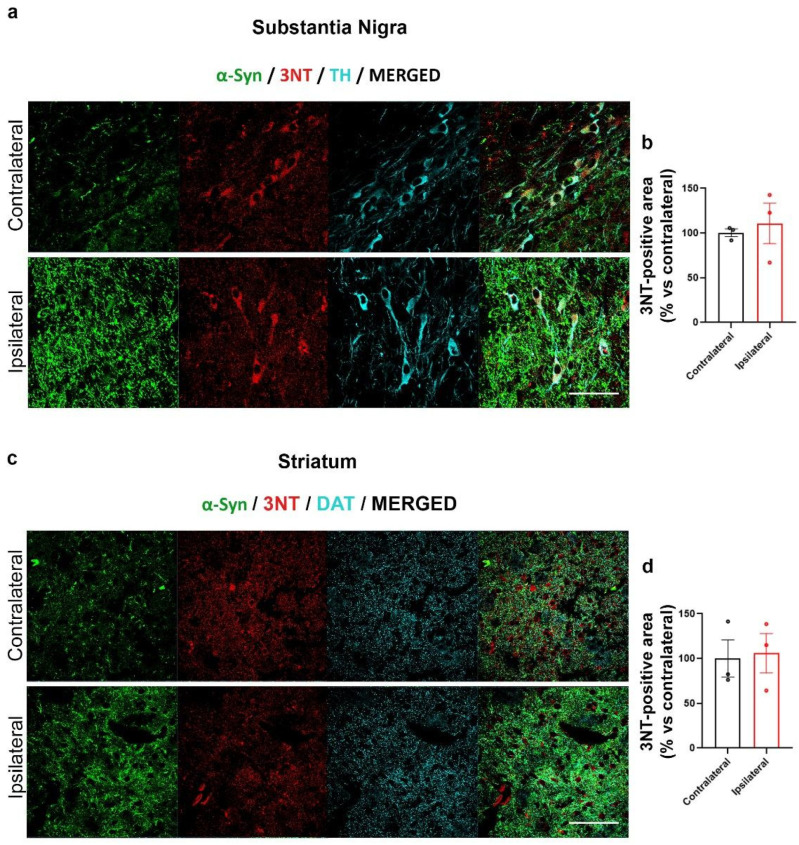
AAV-hα-Syn overexpression did not alter 3NT immunolabeling. Analysis of the total area of 3NT in SN and striatum of 4-month-old wt mice injected with AAV-hα-Syn. (**a**,**c**) Representative IF of α-Syn (green), 3NT (red) and TH or DAT (cyan) in the SN (**a**) and the striatum (**c**) of injected mice after 8 weeks of overexpression. Scale bars: 50 μm. (**b**,**d**) Graphs are showing the 3NT-immunopositive signal in the IL SN (**b**) or striatum (**d**) of AAV-hα-Syn injected mice compared to the CL hemisphere. The values of 3NT were normalized on TH or DAT for SN and striatum, respectively. No statistical differences were observed in IL compared to the CL sides upon the overexpression of hα-Syn, N = 3.

In order to understand whether our AAV-based mouse model of PD may exhibit OS, we assessed the presence of 3NT by IF labelling (Figure 4). For the analysis of the SN, three regions of interest (ROIs) surrounding three different TH positive cell bodies including the nucleus were drawn for each image in order to limit the analysis of 3NT to TH-positive cells, while for the analysis in the striatum, we took into consideration the entire area of the images.

**Figure 5:**
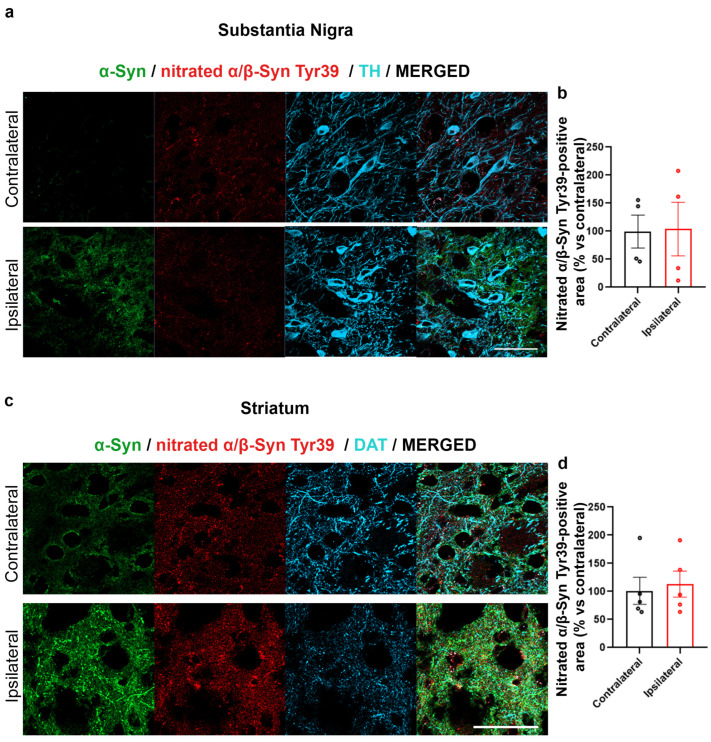
AAV-hα-Syn overexpression did not alter Tyr39 α/β-Syn nitration. Analysis of the total area of nitrated α/β-Syn at Tyr39 in SN and striatum of 4-month-old wt mice injected with AAV-hα-Syn. (**a**,**c**) Representative IF of α-Syn (green), nitrated α/β syn at Tyr39 (red) in the SN (**a**) and striatum (**c**) of injected mice after 8 weeks of overexpression. Scale bars: 50 μm. (**b**,**d**) Graphs are showing the analysis of nitrated α/β-Syn at Tyr39-positive area in SN (**b**) and striatum (**d**) normalized on TH or DAT, respectively, expressed as % vs. the respective CL side. The injection of AAV-hα-Syn did not change the levels of nitration at Tyr39 (N = 4–5).

Interestingly, no difference in 3NT immunolabeling was observed between the SN and in striatum IL or CL to AAV-hα-Syn injection (Figure 4a–d). Consistently, nitration on Tyr39 of α-Syn did not change when comparing CL and IL SN and striatum of AAV-hα-Syn-injected C57BL/6J mice (Figure 5a–d). 

**Figure 6:**
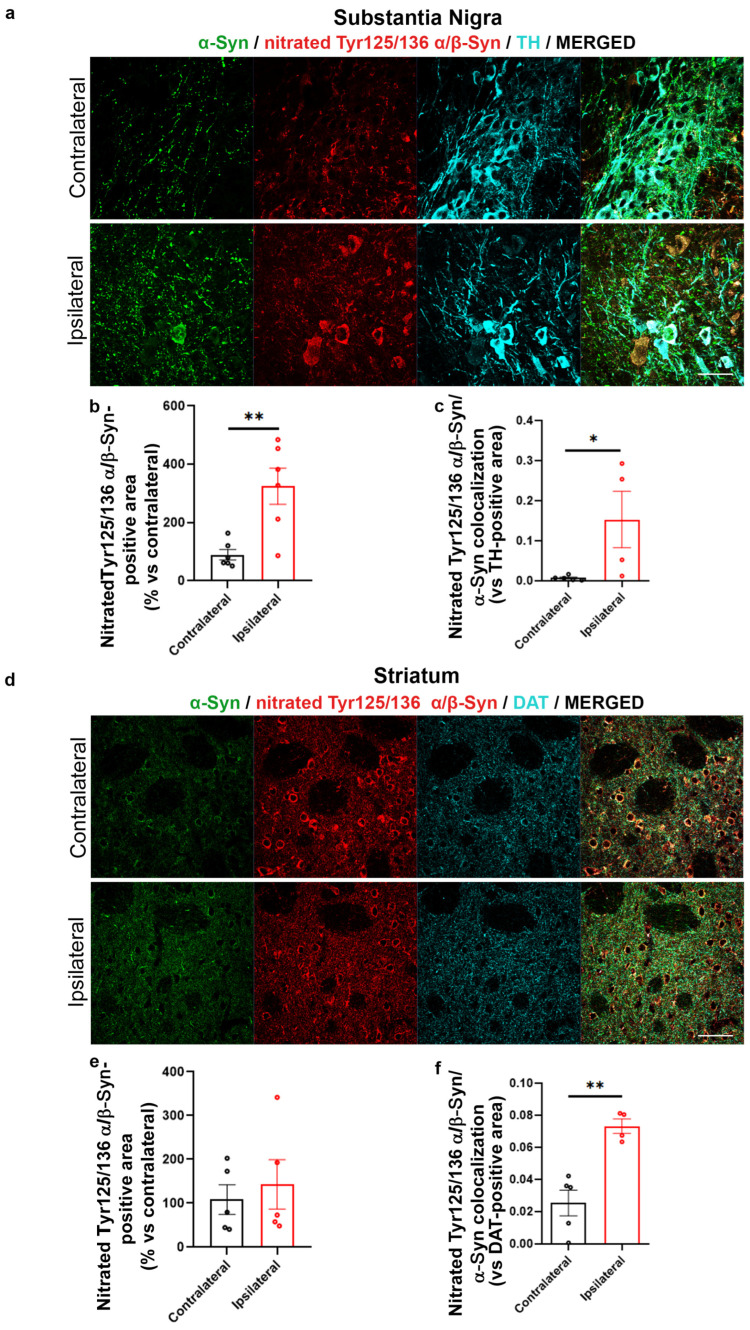
Increased Tyr125/Tyr136 α/β-Syn nitration in the SN and striatum IL to AAV-hα-Syn injection. Representative photomicrographs showing Tyr125 and Tyr136 nitrated α/β-Syn immunolabeling in SN (**a**) and striatum (**d**) of 4-month-old C57BL/6J mice injected with AAV-hα-Syn. Scale bars: 50 μm. (**b**) The graph is showing the analysis of Tyr125 and Tyr136 nitrated α/β syn immunolabeling vs. TH-immunopositive area in the IL SN compared to the CL side (mean difference: +224.8% ** *p* < 0.01; unpaired Student’s *t*-test, N = 5–6). (**c**) The graph represents the area of colocalization between α-Syn and nitrated α/β-Syn at Tyr125 and Tyr136 in SN normalized on TH. A statistical increase was observed in the IL SN when compared to CL hemisphere (mean differences: +0.143, * *p* < 0.05. Student’s unpaired *t*-test, N = 4–5). (**e**) The graph shows the analysis of Tyr125 and Tyr136 nitrated α/β-Syn immunolabeling vs. DAT-immunopositive area in the IL SN compared to the CL side. (**f**) The graph represents the area of colocalization between α-Syn and nitrated α/β-Syn at Tyr125 and Tyr136 in striatum normalized on DAT. A statistical increase was observed in the IL striatum when compared to CL hemisphere (mean differences: +0.048, ** *p* < 0.01. Student’s unpaired *t*-test, N = 4–5).

Notwithstanding, we found an unexpected significant increase in the signal immunopositive for nitrated α/β-Syn at Tyr125 and Tyr136 in the AAV-hα-Syn-injected SN vs. the CL side (Figure 6a,b). Of note, this was accompanied by a significant increase in total α-Syn and nitrated Tyr125/136 α/β-Syn colocalization that was supportive of the fact that these Tyr nitrations mainly involved α-Syn (Figure 6b) rather than β-Syn. In parallel, although we still found a significant increase in total and nitrated Tyr125/136 α/β-Syn colocalization in the IL SN, we did not detect differences between the total immunoreactivity of Tyr125/136 α/β-Syn between the striatum IL and CL to AAV-α-Syn injection. These findings support that human α-Syn overexpression drives selective changes in Tyr nitration in the SN, but this alteration does not occur as a consequence of overall protein 3NT modifications.

### 2.3. Increase of Lys Acetylation in the AAV-hα-Syn-Based Mouse Model of PD

**Figure 7:**
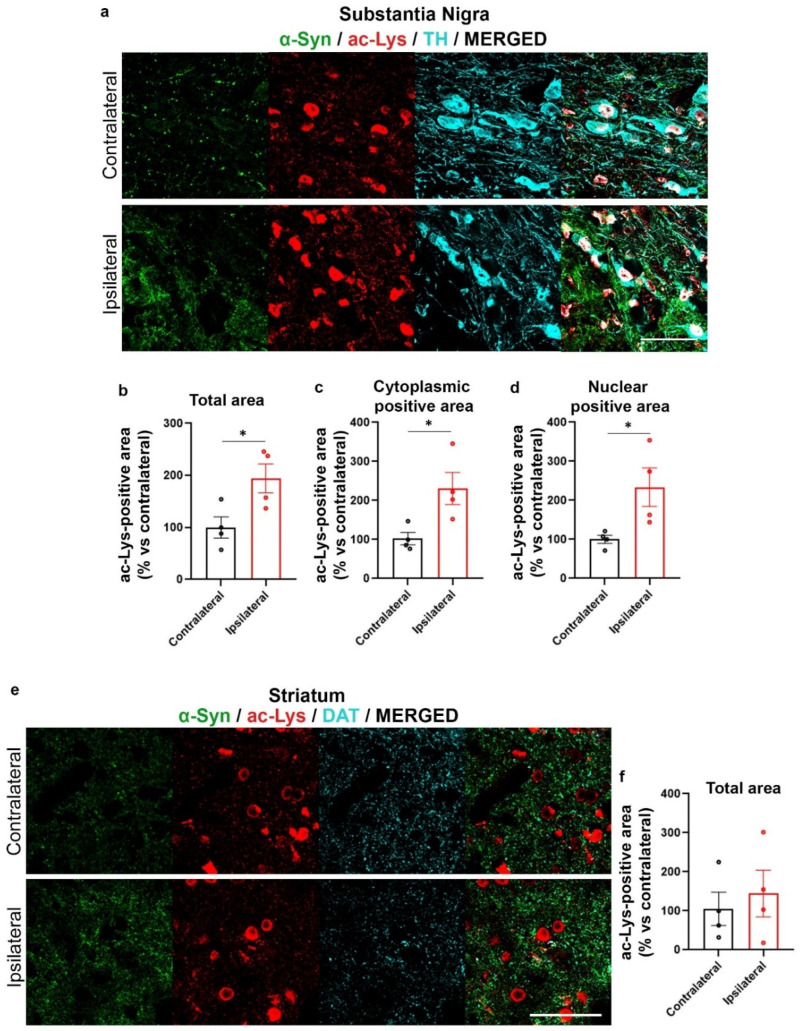
Increased protein acetylation in the AAV-hα-Syn-injected SN. Analysis of the total area of ac-Lys in SN and striatum of 4-month-old wt mice injected with AAV-hα-Syn. (**a**,**e**) Representative IF images of α-Syn (green), ac-Lys (red) and TH (cyan) in the injected SN and of α-Syn (green), ac-Lys (red) and DAT (cyan) in the striatum IL to AAV-hα-Syn injection. Scale bars: 50 μm. (**b**) Graph is showing the analysis of the total ac-Lys-immunopositive area in the IL and CL SN of AAV-hα-Syn mice. Please note the significant increase of ac-Lys-positive area in the IL vs. CL SN (mean difference: +94.42% * *p* < 0.05 Student’s unpaired *t*-test). (**c**) Cytoplasmic ac-Lys-positive area was increased in the IL SN compared to the CL side (mean difference: +130.84% * *p* < 0.05 Student’s unpaired *t*-test, N = 4). (**d**) Nuclear ac-Lys area was significantly augmented in the IL SN compared to the CL side (mean difference: +133.86% * *p* < 0.05 Student’s unpaired *t*-test, N = 4). (**f**) No statistical difference in ac-Lys total area was observed in IL compared to the CL sides upon the overexpression of hα-Syn in the striatum (N = 4).

We then found that overall Lys acetylation was increased in AAV-hα-Syn-injected SN (Figure 7a,b), but not in the IL striatum (Figure 7e,f). As acetylation is important in the control of nuclear functions, the acetylated Lys (ac-Lys) signal localizing in the TH-positive neuron cytoplasm and in cell nuclei was analyzed separately. We found that in the SN, the overexpression of α-Syn induced an over-deposition of ac-Lys both in the cytoplasm and nucleus of TH-positive cells (Figure 7c,d). 

**Figure 8:**
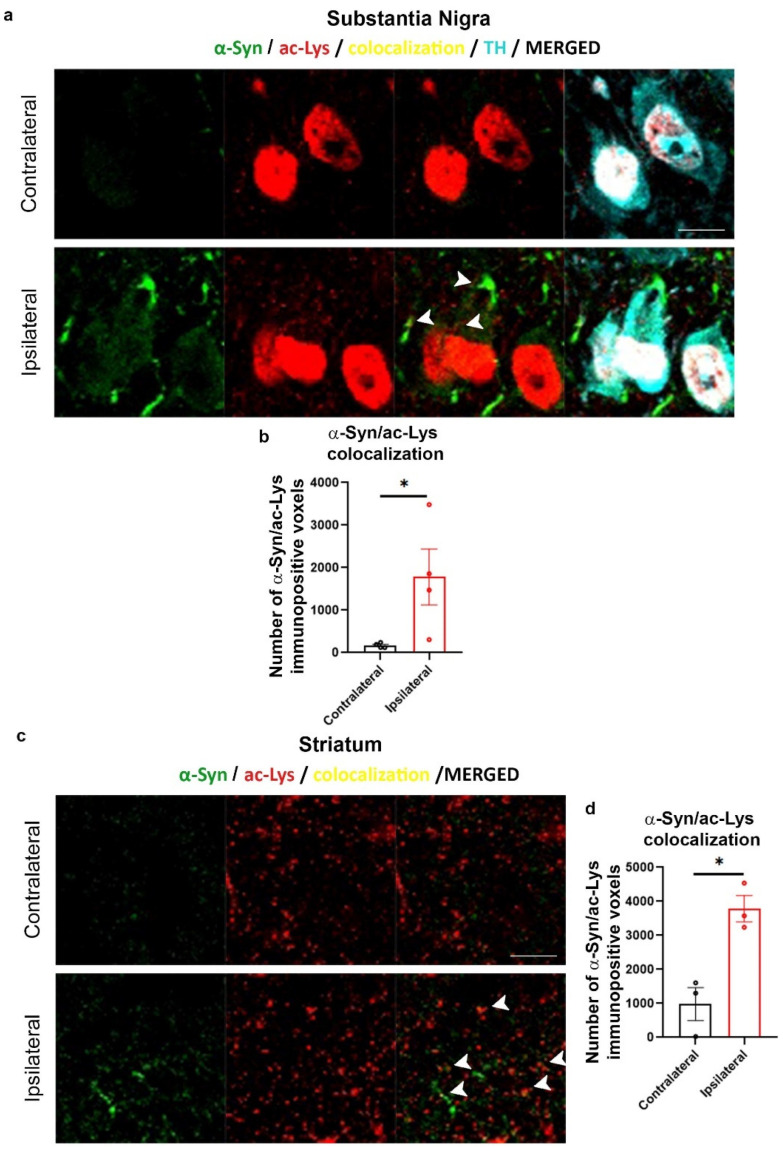
Increased α-Syn/ac-Lys colocalization in the SN and striatum IL to AAV-hα-Syn injection. Analysis of the area of colocalization of the ac-Lys- and α-Syn-positive signals in the SN and striatum of 4-month-old wt mice injected with AAV-hα-Syn. (**a**,**c**) Representative IF of α-Syn (green), ac-Lys (red) and TH (cyan) in the SN and striatum of injected mice after 8 weeks of overexpression. Scale bars: 10 μm. The arrowheads represent α-Syn and ac-Lys colocalization in the SN and striatum IL to AAV-hα-Syn injection. (**b**,**d**) Graphs are showing the analysis of the colocalization area in the IL SN (**b**) or striatum (**d**) of AAV-hα-Syn mice that increased when compared to the CL side (mean difference: +1611 voxels in the SN, +2806 voxels in the striatum, * *p* < 0.05 Student’s unpaired *t*-test, N = 3–4).

When we then analyzed the colocalization between the α-Syn- and ac-Lys-positive signals and we also found a significant increase in the number of α-Syn- and ac-Lys colocalizing voxels both in the IL SN (Figure 8a,b) and striatum (Figure 8c,d) of AAV-hα-Syn-injected mice when compared to CL hemisphere, supporting an augment of Lys-acetylated α-Syn.

## 3. Discussion

The results of this study support that the main α-Syn PTMs detected in the pathological aggregates found in PD brains, Ser129 phosphorylation, nitration and acetylation, are recapitulated in the AAV-based α-Syn overexpression mouse model of PD.

First, we analyzed pα-Syn129 in the SN as this PTM has already been described in mice with nigrostriatal AAV-based α-Syn overexpression [56,59]. Interestingly, we found a mild accumulation of pα-Syn129 in the SN IL to AAV-hα-Syn injections, that by taking into account our previous observations showing a marked pα-Syn129 increase in the striatum IL to the AAV-hα-Syn injection [56], supports that this PTM mainly involves dopaminergic striatal terminals and processes in the initial phases of α-Syn accumulation. This appears consistent with recent findings showing that pα-Syn129 mainly occurs at synaptic terminals, as it is important for regulating α-Syn synaptic function and is promoted by neuronal activity [24]. However, it is relevant to mention that other studies demonstrated that pα-Syn129 occurs subsequently to initial α-Syn aggregation and can in turn reduce α-Syn aggregation propensity and toxicity [20]. Since we previously described that AAV-hα-Syn-injected mice exhibit a marked striatal and nigral deposition of insoluble fibrillary α-Syn deposits, striatal synaptic vesicle rearrangements and dopamine release deficits as well as dopaminergic nigral neuron loss at the same time point analyzed in this study [56], it sounds feasible that the detected increase in Ser129 phosphorylation occurred as a consequence of α-Syn aggregates accumulation. Nevertheless, we cannot definitely rule out the possibility that the initial AAV-mediated increase of soluble human α-Syn may have affected neuronal activity and in turn may have stimulated Ser129 α-Syn phosphorylation [24].

We then observed that, despite a general trend toward the increase in the 3NT-immunopositive signal, the differences in 3NT levels between the SN and striatum CL and IL to AAV-hα-Syn injection were not significant, supporting that human α-Syn overexpression did not induce a significant OS. However, though we did not detect an increase in Tyr39-nitrated α-Syn-immunopositive area in the SN or striatum IL to AAV-hα-Syn injection, we found a significant increase in Tyr125 and Tyr136 nitrated α/β-Syn-positive area, that resulted to mostly colocalize with total α-Syn-immunopositive signal. These findings support that the overexpression of human α-Syn leads to specific Tyr nitration of the protein. Interestingly, these observations appear consistent with previous studies on early PD patient sera, which showed that a specifically enhanced Tyr125 and Tyr136 α-Syn nitration occurs in the absence of overall differences in 3NT levels [60,61]. It is also worth mentioning that Tyr125 nitrated α-Syn displays an increased aggregation speed when compared to Tyr39 nitrated α-Syn [62]. In addition, synthetic fibrils formed by Tyr125-nitrated α-Syn are short, exhibit increased propensity to stack in parallel and are more prone to form aggregates [62]. Other studies showed that nitration of the α-Syn C-terminus, including at Tyr125/136, affect α-Syn conformation by impacting on Tyr cross-linking [63]. This notwithstanding, α-Syn C-terminal nitration does not appear to perturb Ser129 phosphorylation in vitro [62], which sounds also consistent with the present findings showing increased pα-Syn129 in the AAV-hα-Syn overexpression model.

Our results are also supportive of an increase in general protein acetylation, including α-Syn acetylation, upon the AAV-based human α-Syn overexpression. In particular, we detected a significant increase in ac-Lys in both the cytoplasm and in the nuclei of TH positive cells in the AAV-hα-Syn-injected SN and we also observed a significant increase in the colocalization between the ac-Lys- and α-Syn-immunopositive signal, which is consistent with the occurrence of α-Syn acetylation. 

These findings are reminiscent of the increase in general protein acetylation that has been detected in PD patient fibroblasts and can be ascribed to mitophagy dysfunction and damaged mitochondrial accumulation, in turn, decreasing sirtuin activity through the impairment of NAD+ generation [64,65]. In addition, a significant increase in full length acetylated α-Syn in the LB-enriched and SDS-insoluble protein fractions from the brain of PD patients has also been reported [66,67] and this imbalance in protein acetylation has been hypothesized to contribute to PD pathogenesis [68]. Remarkably, an accumulation of nuclear pα-Syn129 has also been detected in the post-mortem brains of PD patients, hinting that an increase in this posttranslationally modified form of the protein can very well impact on α-Syn nuclear functions [69]. This is also in agreement with recent findings showing that α-Syn can enter in the nucleus where it regulates gene transcription and promotes DNA repair, while its pathological cytoplasmic aggregation reduces its nuclear levels, thus contributing to cell death via nuclear damage [70,71]. Still, alterations in α-Syn nuclear functions have been also reported in induced pluripotent stem cells-derived neurons from PD patients with SNCA multiplications [72]. Our findings also support the occurrence of an increase in α-Syn acetylation in the striatum, suggesting that this phenomenon may have affected α-Syn-mediated control of synaptic vesicles.

Indeed, acetylation mainly occurs in the N-terminal region of α-Syn, which is important for the interaction with biological membranes. Consistently, acetylated α-Syn has been found to exhibit altered ability to interact with synaptic vesicle mimics [14,73,74,75]. It may thus be feasible that an alteration in α-Syn-mediated synaptic vesicle control following acetylation could be at the basis of the synaptic damage that we previously reported in AAV-hα-Syn mice [56]. Moreover, since in eukaryotes N-terminal α-Syn acetylation occurs co-translationally, it has been hypothesized that it precedes all other PTMs, which is confirmed by the fact that non-acetylated α-Syn is less phosphorylated on Ser129 [76]. This suggests that another possible mechanism through which α-Syn acetylation can affect synaptic function is also the control of Ser129 phosphorylation, which is pivotal for synaptic activity [24].

In relation with the role of α-Syn acetylation, it has been also found that SIRT2 removes acetyl groups from α-Syn, while the inhibition of SIRT2 decreases α-Syn toxicity in in vitro and in vivo models of synucleinopathy [49,52]. This supports that acetylation limits the toxic potential of α-Syn. Moreover, N-terminal acetylation of α-Syn can negatively impact on α-Syn aggregation behavior [12,13,77]. It may thus be also feasible that the increase in general protein acetylation, which also appears to involve α-Syn in the AAV-based overexpression model, may play a relevant role in limiting the rate of α-Syn aggregation. This would also fit with evidences showing that Ser129 phosphorylation, that is increased in acetylated α-Syn, also decreases the aggregation propensity of the protein [20].

Taken together, our observations support that the overexpression of AAV-hα-Syn in the nigrostriatal system recapitulates the main α-Syn PTMs observed in the post-mortem brain of PD. In particular, they suggest that in the initial phases of α-Syn, pathological aggregation Ser129 α-Syn phosphorylation and acetylation can be both detected at striatal synaptic terminals, while nigral neuronal cell bodies show a specific accumulation of Tyr 125/136 nitrated α-Syn in the absence of Tyr39 nitration and 3NT changes as well as a general nuclear and cytoplasmic increase in acetylated proteins including α-Syn.

Further studies will be necessary in order to better characterize the significance of these findings. For instance, we are aware that we will need to expand this study to female mice in order to take into account the differences in pathological mechanisms, clinical outcomes and response to treatments that have been detected between male and female PD patients [78,79]. In addition, since the accumulation of α-Syn is not solely restricted to dopaminergic neurons, it would be interesting to assess the effect of α-Syn accumulation in other brain areas by using more appropriate experimental models.

Nevertheless, our findings indicate that the AAV-hα-Syn-based mouse model of PD represents a valuable experimental platform to investigate the role of α-Syn PTM as well as their impact on α-Syn aggregation and on neuronal function and toxicity. In light of the fact that α-Syn PTMs are emerging as possible therapeutic targets to cure PD [15], their characterization in experimental models may help the development of novel approaches to slow down disease progression. Moreover, by expanding our understanding on the occurrence and progression of α-Syn PTM, the result of this study can implement our knowledge on the biological basis of PD and enable a better interpretation of the results from the multitude of toolkits that have been developed to assess whether α-Syn PTM can be considered as potential biomarkers for disease progression and differential diagnosis [60,80,81,82,83,84,85,86,87,88].

## 4. Materials and Methods

### 4.1. AAV-hα-Syn Overexpression in Mice

AAV injections were performed on 2-month-old C57BL/6J wt male mice (Charles River, Wilmington, MA, USA). Animals were bred at the University of Brescia animal house facility and maintained under a 12 h light–dark cycle at a room temperature (rt) of 22 °C and had ad libitum food and water. All experiments were made in accordance with Directive 2010/63/EU of the European Parliament and of the Council of 22 September 2010 on the protection of animals used. All experimental and surgical procedures conformed to the National Research Guide for the Care and Use of Laboratory Animals and were approved by the Animal Research Committees of the University of Brescia (Protocol Permit 719/2015-PR). All achievements were made to minimize animal suffering and to reduce the number of animals used. The plasmids for the production of AAV serotype 2/6 inducing the overexpression of either human wt α-Syn, and GFP (AAV-GFP), driven by the Synapsin I promoter and enhanced using a woodchuck hepatitis virus posttranscriptional regulatory element (WPRE), were produced as previously described [54,55] and were acquired by the University of Lund, Sweden or Vector Biolabs. Two-month-old (25 g) wt mice were injected in the left SN with AAV-hα-Syn or AAV-GFP as control diluted at 5 × 10^13^ genome copies/mL.

Briefly, animals were anesthetized and placed in a stereotactic head frame (Stoelting, IL, USA). After making a midline incision of the scalp, a hole was drilled in the appropriate location for the SN. Two microliters of viral vector were injected at a rate of 0.2 µL/min with a 33-gauge needle on a 10 µL Hamilton syringe at the following coordinates: antero-posterior −3.60; medio-lateral +1.15; dorsal-ventral −3.75 relative to Bregma. The needle was left in place for an additional 5 min before being slowly retracted from the brain. After 8 weeks of overexpression of the virus, mice were transcardially perfused with Immunofix in order to keep the fixed tissues for the IF analysis.

### 4.2. Immunofluorescence Labeling of Mouse Tissues

To perform IF labeling, mice were anesthetized with chloral hydrate 400 mg/kg i.p. (Sigma-Aldrich, St. Louis, MO, USA) and transcardially perfused with ice-cold Immunofix. Brains were post-fixed for 4 h in Immunofix and conserved in 18% sucrose in PBS 0.1 M. Coronal sections of 25 μm containing the substantia nigra and striatum were cut with a cryostat and conserved in 60% glycerol. After permeabilization in PBS 0.1 M supplemented with 20% methanol and 0.3% Triton X-100, free floating slices were incubated for 2 h at room temperature (rt) in strong blocking solution (10% Normal Goat Serum (NGS), 3% Bovine Serum Albumin (BSA), 0.05% NaN_3_, 0.3% Triton-X100 in PBS 0.1 M) and then with the primary antibody in blocking solution overnight at 4 °C. The following day, slices were washed with 0.3% Triton-X100 PBS 0.1 M and incubated with the fluorochrome-conjugated secondary antibody in 0.3% Triton-X100 PBS 0.1 M plus 1 mg/mL BSA for 1h at rt. After three washes in 0.3% Triton X-100 PBS, slices were incubated for 2 h at rt with the second primary antibody prepared in blocking solution, followed by incubation for 1 h at rt with the optimal fluorochrome-conjugated secondary antibody. The sections undergoing triple immunolabeling were further subjected to a third incubation with a third primary antibody for 2 h at rt followed by incubation for 1 h with the fluorochrome-conjugated secondary antibody. Finally, the slices were mounted onto superfrost slides using Vectashield mounting medium for fluorescence (Vector Laboratories, Mowry Ave Newark, CA, USA).

### 4.3. Antibodies

Table 1 summarizes the antibodies used for IF analysis and the respective working concentrations. The secondary antibodies for IF were goat anti-mouse/rabbit/rat IgG Cy3-conjugated, Alexa488-conjugated, Alexa647-conjugated or goat anti-mouse/rabbit biotinylated plus Streptavidin 594 (Jackson ImmunoResearch, Milan, Italy).

### 4.4. Confocal Microscopy

For confocal analysis, samples were observed by LSM880 Zeiss confocal laser microscope (Carl Zeiss S.p.A., Milan, Italy). The height of sections scanning was 1 μm. Images (512 × 512 pixels) were then reconstructed using ZEISS ZEN Imaging Software (Carl Zeiss S.p.A.). The acquisition parameters during confocal imaging were maintained constant for all the images acquired to perform the analysis.

### 4.5. Image Analysis

Analysis of α-Syn, pα-Syn129, 3NT, nitrated α/β-Syn at Tyr39, nitrated α/β-Syn Tyr125/136, ac-Lys, DAT and TH-immunopositive area in the SN and striatum was performed by quantifying the diverse immunoreactivities in the SN and striatum on the digitized images using the FIJI (NIH) software (ImageJ 1.53t). Brains from different mice (10 sections from each mouse, two every 150 µm) were analyzed by examining an average of 4–5 fields per section. 

In some cases, in the striatum we drew regions of interest (ROIs) in order to exclude the signal from cholinergic or GABAergic cell bodies. For SN, we used the TH positive signal to outline the cell bodies and exclude the nuclei in order to analyze the specific immunostaining. To this purpose, for each image, we identified 3 representative ROIs on which we did the statistical analysis.

### 4.6. Analysis of the Area of Colocalization of Immunopositive Signals

In order to understand whether two fluorescent signals colocalized in the same image, we used the Colocalization Tools in ZEN Blue (Blue edition v3.4, Carl Zeiss Microscopy GmbH, Oberkochen, Germany). Throughout the same experiment, we applied the same threshold for each channel, in order to take into consideration the same number of pixels. Eventually, by exploiting the data table offered by the software, we noted the values of total scaled area (μm^2^) of Pixel Count, representative for the area of colocalization of the two signals.

### 4.7. Statistical Analysis

Statistical differences in each experiment were assessed using Student’s unpaired *t*-test with statistical significance established at *p* < 0.05. All data are presented as mean ± SEM. The numbers of animals used for each experimental group in the different experimental studies ranged between N = 3–9.

## Figures and Tables

**Table 1:** List of primary antibodies.

Primary Antibodies	Company	Working Concentration	Catalogue Number
α-Syn (SYN1)	BD	1:500	610787
pα-Syn129	Invitrogen	1:500	PA5-37740
3NT	Millipore	1:400	06-284
Nitrated α/β-Syn Tyr125/136	Millipore	1:500	36-011
Nitrated α/β-Syn Tyr39	Millipore	1:500	36-012
ac-Lys	Cell Signalling	1:500	9441
TH	Millipore	1:500	AB152
DAT	Santa Cruz	1:300	sc-32258

## Data Availability

The data presented in this study are available on request from the corresponding author. The data are not publicly available due to intellectual property restrictions.
